# Left Ventricular Global Longitudinal Strain in Patients With COVID-19 Infection

**DOI:** 10.7759/cureus.23986

**Published:** 2022-04-09

**Authors:** Harneet Bhatti, Andres Cordova Sanchez, Rajat Dhungana, Christos Carvounis, Avneet Singh

**Affiliations:** 1 Internal Medicine, State University of New York Upstate Medical University, Syracuse, USA; 2 Cardiology, State University of New York Upstate Medical University, Syracuse, USA

**Keywords:** left ventricular function, transthoracic echocardiography, left ventricular global longitudinal strain, myocardial strain, covid-19

## Abstract

Coronavirus disease 2019 (COVID-19), caused by the severe acute respiratory syndrome coronavirus 2, is an ongoing pandemic that has affected millions globally. Many infected patients have been noted to have cardiovascular damage. Prior to the development of clinical symptoms, the use of transthoracic echocardiography, specifically with measurements of left ventricular global longitudinal strain (LVGLS), may provide an additional prognostic marker for patients infected with COVID-19. We sought to determine whether patients with COVID-19 and reduced LVGLS have an increased risk for mortality. The mean LVGLS was determined to be significantly lower in the non-survivors compared to the survivors (−11.6 ± 1.8 vs −15.4 ± 0.74, p<0.05). It should be noted, however, that even those that survived were found to have reduced LVGLS (<−18.5%). A multivariate logistic regression analysis was also performed that demonstrated a relationship between reduced LVGLS and an increased risk for mortality. Overall, our data indicate that COVID-19 patients may have subclinical left ventricular dysfunction, and that critically ill patients may have a greater decline in cardiac dysfunction.

## Introduction

Coronavirus disease 2019 (COVID-19), which is caused by the severe acute respiratory syndrome coronavirus 2 (SARS-CoV-2), is an ongoing pandemic that has affected millions globally. Although the most recognized clinical manifestations of COVID-19 are respiratory symptoms, some patients have been found to have cardiovascular damage without any clinical cardiac findings [1-5]. Studies have revealed that virus particles are present in the myocardium and vascular endothelium in patients with COVID-19. Therefore, it is necessary to study the long-term consequences on the cardiac function of patients infected with SARS-CoV-2 [6,7]. Transthoracic echocardiography (TTE) remains an important imaging modality used for assessing cardiac function, which can provide relevant information that may impact clinical management for those infected with SARS-CoV-2 [8]. Left ventricular global longitudinal strain (LVGLS), measured by speckle tracking echocardiography (STE), provides a way to quantify myocardial deformation independent of left ventricular ejection fraction (LVEF) [9]. It has both diagnostic and prognostic capabilities and has known clinical applications in patients with left ventricular hypertrophy, valvular diseases, and cardiomyopathies. The reduction of absolute strain is known to signify a poor prognosis for some myocardial diseases and may signify a poor prognosis in other settings as well. Recent studies demonstrated that both left ventricular global longitudinal strain from the apical four-chamber views (LVGLS4CH) and right ventricular free wall longitudinal strain were predictors of mortality in patients with COVID-19 [10,11]. Additional studies, however, suggest that only decreased right ventricular strain may be associated with poor prognosis in patients with COVID-19 [12,13]. On the other hand, another study determined that reduced LVGLS was not associated with poor outcomes [14]. In this retrospective study, we sought to determine whether reduced LVGLS in patients with COVID-19 is associated with an increased risk for mortality.

## Materials and methods

This is a retrospective study conducted at the State University of New York (SUNY) Upstate Medical University, Syracuse, NY, USA. The patients selected for this study were those that were >18 years of age, tested positive for SARS-CoV-2 using the reverse transcriptase-polymerase chain reaction (PCR) assay from nasopharyngeal swabs between April 1st, 2020 and December 1st, 2020, were hospitalized for COVID-19 related symptoms, and had a TTE during that hospitalization. Data were collected through chart review. Along with demographic data, troponin T levels on admission, peak troponin T levels, and pro-B-type natriuretic peptide (proBNP) levels were also obtained.

**Figure 1:**
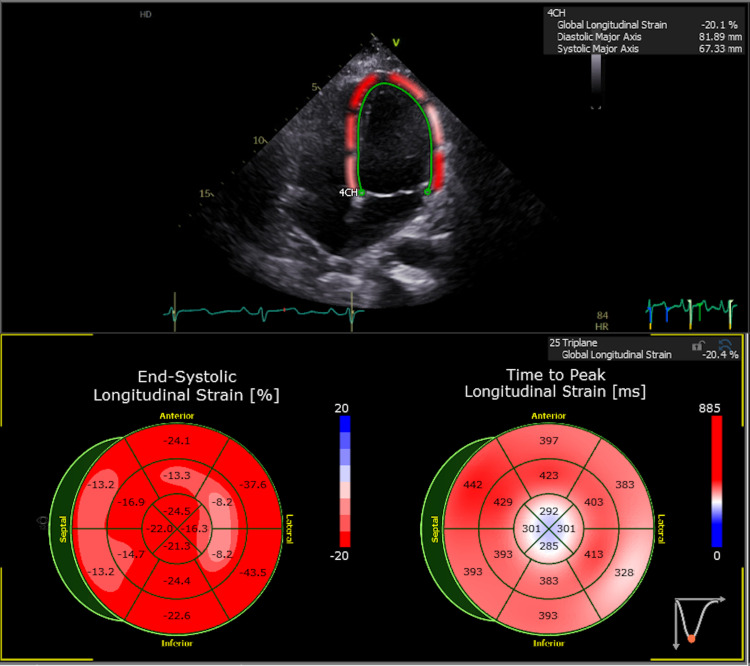
Example of LVGLS evaluation. 2D speckle tracking analysis demonstrated that the patient had a LVGLS of −20.4%. LVGLS: left ventricular global longitudinal strain.

Patients underwent TTE using the Vivid echocardiography system (GE Healthcare, Chicago, IL, USA). Only patients with optimal apical two-, three-, and four-apical chamber views were included in this study. Measurements of LVGLS were obtained via speckle tracking using Centricity Cardio Workflow V7.0 (GE Healthcare, Chicago, IL, USA). Two-, three-, and four-chamber views were used to measure LVGLS. The software provided automated detection of the LV endocardial boundary, which was then edited, if needed, to conform to the visualized LV boundaries. The maximum negative value of strain during systole, as measured by the software, represented the maximum contractility for each segment. The average of these values from each segment was used to determine LVGLS. LVGLS was considered to be abnormal if <−18.5%. An example of LVGLS determination using STE is demonstrated in Figure 1. One user-determined LVGLS for all patients included in this study to limit variability. The endpoint evaluated in this study was mortality during hospitalization.

Continuous numeric variables were expressed as mean ± standard deviation (SD) and compared using a two-sample Student’s t-test in the two groups of patients: survivors and non-survivors. The Chi-square was used to compare the frequency of nominal data in the two groups. A multivariate logistic regression analysis was also performed to assess any predictors of mortality. A P-value <0.05 was considered statistically significant.

## Results

A total of 90 patients met the selection criteria for this study. Forty-two of these patients had to be excluded given poor image quality on TTE and, consequently, an inability to determine LVGLS. Thus, 48 patients were included in this study and were divided into two groups: survivors and non-survivors. A total of 37 patients survived and were discharged, while 11 died during hospitalization.

There was a significant difference in the age of the patients that survived compared to those that did not (Table 1). The non-survivors tended to be older, with a mean age of 76.5 years. There was no significant difference in medical comorbidities, including diabetes mellitus, hypertension, hyperlipidemia, coronary artery disease, and end-stage renal disease, between the two groups. The mean LVGLS was determined to be significantly lower in the non-survivors compared to the survivors (−11.6 ± 1.8 vs −15.4 ± 0.74, p<0.05) (Table 1). It should be noted, however, that even those that survived were found to have reduced LVGLS (< −18.5%). Specifically, 81.3% of all patients, 91% of non-survivors, and 78.4% of survivors had reduced LVGLS. LVEF from TTE during hospitalization was also evaluated, and no significant difference between the two groups was found (Table 1).

**Table 1:** Demographic and clinical characteristics of patients. SD: standard deviation, DM: diabetes mellitus, HTN: hypertension, HLD: hyperlipidemia, CAD: coronary artery disease, ESRD: end-stage renal disease, proBNP: pro-B-type natriuretic peptide, LV: left ventricular, EF: ejection fraction, LVGLS: left ventricular global longitudinal strain, NS: no significant difference between groups. *Thirteen patients were not included as levels were not obtained during hospitalization.

	Total (n=48)	Survivors (n=37)	Non-survivors (n=11)	Significance
Age (years) (SD)	62.9 (15.2)	58.9 (2.35)	76.5 (2.78)	p<0.001
Sex				
Male	17	12	5	
Female	31	25	6	
DM, n (%)	10 (20.8)	9 (24.3)	1 (9.1)	NS
HTN, n (%)	28 (58.3)	22 (59.5)	6 (54.5)	NS
HLD, n (%)	17 (35.4)	13 (35.1)	4 (36.4)	NS
History of CAD, n (%)	5 (10.4)	4 (10.8)	1 (9.1)	NS
ESRD, n (%)	2 (4.2)	2 (5.4)	0	NS
Initial troponin T, mean	0.23 (0.39)	0.05 (0.13)	0.22 (0.44)	p<0.05
Peak troponin T, mean	0.26 (0.43)	0.04 (0.15)	0.24 (0.49)	p<0.05
proBNP, mean (SD)	3,551	824 (196)*	9,503 (4,081)	p<0.005
Mildly reduced LV function (EF 40-50%), n (%)	4 (8.3)	3 (8.1)	1 (9.1)	NS
Moderately reduced LV function (EF 30-39%), n (%)	2 (4.2)	0	2 (18.2)	NS
Severely reduced LV function (EF <30%), n (%)	0	0	0	
Respiratory failure requiring intubation, n (%)	5 (10.4)	4 (10.8)	1 (9.1)	NS
LVGLS, mean (SD)	−14.53	−15.4 (0.74)	−11.6 (1.8)	p<0.05

Pro-B-type natriuretic peptide (proBNP) levels were found to be elevated in 80% of all patients, and there was a significant difference seen between the two groups. Non-survivors had significantly higher levels of proBNP when compared to the survivors (Table 1). In addition, troponin T levels were elevated in 63.6% of the non-survivors and in 24.3% of the patients that survived (p<0.05).

A multivariate logistic regression analysis was performed to identify any association between the markers studied and mortality. A significant relationship was identified that included age, proBNP, and LVGLS, while the other markers were not effective. Although rates of abnormal troponin T levels were significantly higher in those that did not survive, this marker did not significantly influence the risk of mortality. The relationship was determined to be Logit P = −11.67 + (0.13 × age) + (0.000457 × proBNP) − (0.043 × LVGLS), likelihood ratio test statistic = 20.13 (p<0.001). Age was the dominant factor discriminating between survivors and non-survivors. However, it should be mentioned that the two remaining markers were of significance as indicated by their relationship to mortality when age was removed. In that case, Logit P = −0.73 + (0.0006 × proBNP) + (0.08 × LVGLS), likelihood ratio test statistic = 10.74, (p = 0.005). Among these two markers, the relationship between mortality and proBNP was stronger. Nonetheless, it should be noted that there is a strong relationship between proBNP and LVGLS with proBNP = 14,682 + (784 (LVGLS)), r = 0.51, r2 = 0.26, p<0.001.

Only 14.6% of all patients had normal proBNP levels, of which 85.7% had reduced LVGLS. About 16.7% of patients with normal proBNP and reduced LVGLS did not survive, whereas 83.3% of patients with normal proBNP and reduced LVGLS survived.

## Discussion

Myocardial injury in COVID-19 patients can occur in many ways. There may be direct myocardial injury from viral infiltrates, or there may be indirect injury from increased respiratory distress and resultant hypoxemia, or from the immune response and systemic hyperinflammation [15]. Evidence of myocardial injury was seen through increased troponin T levels in patients that did not survive, indicating that severe symptoms may lead to complications involving cardiac insults. In addition, non-survivors were noted to have significantly higher levels of proBNP, further suggesting that cardiac dysfunction in COVID-19 patients may contribute to poor outcomes. Evidently, a regression analysis demonstrated that elevated proBNP levels were associated with an increased risk for mortality.

In this study, a significant reduction in LVGLS was seen in non-survivors, whereas no significant difference in LVEF was found. Interestingly, even those patients with COVID-19 that survived had on, average, decreased LVGLS. It should be noted, however, that a greater number of the non-survivors were noted to have reduced LVGLS. The mean LVGLS was significantly lower in the non-survivors as well. These data indicate that COVID-19 patients may have subclinical left ventricular dysfunction and that those that are critically ill may have a greater decline in cardiac dysfunction. These data are consistent with other studies that demonstrated a reduction in LVGLS in COVID-19 patients [16,17]. In addition, we found that a reduced LVGLS was associated with an increased risk for mortality.

A considerable number of patients in this study that did survive had reduced LVGLS. Even patients with normal proBNP levels were found to have reduced LVGLS. Thus, it may be important to follow COVID-19 patients that survive for cardiac dysfunction in the future. The long-term effects of COVID-19 are unknown, but given the current collection of information we have, it is not unreasonable to anticipate cardiovascular complications in patients that have been infected with SARS-CoV-2. Further studies determining whether LVGLS improves after recovery and discharge may help determine whether clinical follow-up of cardiac function would change clinical management.

There are several limitations to this study that should be discussed. This includes a small sample size that resulted from the exclusion of patients without adequate TTE images. This may have caused the exclusion of patients that had significant COVID-19 disease, a large body habitus, or limited body positioning. However, the rate of mortality was similar in the studied patients and excluded patients (22.9% vs 23.3%), arguing against significant bias in our study. In a study conducted by Xie et al., it was determined that there was no significant difference between LVGLS and LVGLS4CH [10]. Given the challenges technicians face in obtaining multiple apical views in such patients, future studies may benefit from studying LV GLS4CH. In addition, our study was conducted with one user determining LVGLS, limiting variability among the patients included in this study, but variation in LVGLS among different users may be expected. It should be noted, however, that studies have found that measurements of LVGLS are reproducible even across multiple readers, supporting the generalizability of this study [18-20].

## Conclusions

In conclusion, our results demonstrate that myocardial dysfunction is common in COVID-19 patients. A significant decrease in LVGLS and an increase in proBNP were seen in those that did not survive, and it was found that non-survivors tended to have lower LVGLS. Such findings demonstrate that LVGLS may be useful in determining the prognosis of COVID-19 patients. In addition, a considerable number of patients that survived were noted to have reduced LVGLS. Thus, for those that survive, continued cardiac function monitoring may be warranted.
